# Design and Manufacturing Method of Fundamental Beam Mode Shaper for Adapted Laser Beam Profile in Laser Material Processing

**DOI:** 10.3390/ma12142254

**Published:** 2019-07-13

**Authors:** Christian Bischoff, Friedemann Völklein, Jana Schmitt, Ulrich Rädel, Udo Umhofer, Erwin Jäger, Andrés Fabián Lasagni

**Affiliations:** 1 Topag Lasertechnik GmbH, Nieder-Ramstädter-Str. 247, 64285 Darmstadt, Germany; 2 Institut für Fertigungstechnik, Technische Universität Dresden, George-Bähr-Str. 3c, 01069 Dresden, Germany; 3 Institut für Mikrotechnologien, Hochschule RheinMain, Am Brückweg 26, 65428 Rüsselsheim, Germany; 4 Fraunhofer-Institut für Werkstoff-und Strahltechnik IWS, Winterbergstr. 28, 01277 Dresden, Germany

**Keywords:** phase shifting element, micro technology, laser processing, beam shaping

## Abstract

Many laser material processing applications require an optimized beam profile, e.g., ring shape or Top-Hat profiles with homogeneous intensity distribution. In this study, we show a beam shaping concept leading to a phase shifting element with binary height profile as well as a very low periodicity with near diffraction limited spot size. Further advantages of so-called Fundamental Beam Mode Shaping (FBS) elements are the simplified handling, and a high efficiency and homogeneity. The calculated height profile of FBS elements are transferred in fused silica substrates using a combination of microlithography technologies, reactive ion etching (RIE) and ion beam etching (IBE). The experiments demonstrated a linear relation between the etching depth after RIE and IBE. The optical evaluation of the manufactured FBS beam mode shaper confirmed the presented concept design.

## 1. Introduction

Nowadays, pulsed solid-state lasers are well established for many micromachining processes. In general, picosecond and femtosecond laser sources deliver ultra-short pulses with low energies, causing low thermal effects, leading to high-precision micro structuring processes that enable the fabrication of structures in the micrometer range. In contrast, nanosecond lasers introduce higher thermal effects, but are available at significantly lower costs. As a consequence, in cost-sensitive markets like series production in semiconductor manufacturing, nanosecond lasers are still mainly used [1].

In addition to the pulse duration, the wavelength and the beam profile also have a strong influence on the quality of the laser ablation process. A single-mode beam with a Gaussian intensity distribution is well-known to be the most convenient laser beam spatial distribution. It preserves its distribution while propagating, and even when focused. Furthermore, this intensity distribution allows excellent focusing, which is limited by the diffraction limit of light. However, the spot area limited by a beam diameter (1/e² level) contains only 86.5% of the laser beam power, and the intensity at the boundary is only 13.5% of the peak intensity [2]. As a consequence, for many applications, the raw Gaussian beam profile of single-mode lasers does not lead to the best result.

Several studies have shown that the use of so-called Top-Hat beam profiles with uniform energy distribution can improve process quality and efficiency, especially in thin-film ablation processing [3,4,5,6]. When a Gaussian beam profile is used, the intensity below the ablation threshold does not contribute to the laser process. It can be absorbed, leading to unwanted heating of the material. With the more homogeneous intensity distribution of the Top-Hat profile, more energy is contributed to the ablation process, leading to less heating of the material and thus providing higher efficiency in the ablation process [3,4,5]. A further advantage of the Top-Hat profiles is given by the lower sensitivity of the ablated spot diameter to variations of pulse energy in comparison using Gaussian beams, leading to a more stable laser process [3]. A uniform irradiance is relevant in a variety of applications, including laser-induced break-down spectroscopy (LIBS) [7], direct laser interference patterning (DLIP) [8,9,10], applications in light detection and ranging (LIDAR) [11], and additive manufacturing (SLM) [12].

In addition to the redistribution of the energy from Gauss to a Top-Hat profile, beam shapers can also change the footprint of the focus spot from round to rectangular or square geometry [3,5]. Such rectangular or square footprints can be advantageous, especially for thin film scribing. Gaussian beam profiles typically generate saw-tooth patterns along the scribing trench, as long as the pulse-to-pulse overlap is lower than 80%. Such saw-tooth patterns are in many cases unwanted, as the peaks of the saw tooth pattern can be sources for micro cracks in the electrical devices which reduce their life time. By using square shaped focus spots, trenches with smooth sidewalls can be achieved. Furthermore, the pulse-to-pulse overlap can be significantly reduced, and process speed will be increased at the same time [4,5,13].

Top-Hat beam shaping optics typically transform an incoming collimated laser beam into a round or square shaped uniform intensity distribution. The homogenization of laser beam intensity is based on refractive or diffractive optics and implemented by integration or deterministic transformation methods. Refractive Top-Hat shapers for coherent Gaussian (TEM00) beams can be designed using classical raytracing or analytical methods based on the principle of conversation of energy [14,15]. With these methods, it is possible to calculate for each point of the input plane a local deflection angle (called “mapping”) to receive the desired profile in the image plane. This results in smooth and continuous surface profiles which can be realized as aspheric or free-form optic. The integration method [14] can be realized using micro lens arrays. Here, the original incoming beam will be divided into several sub-beams which are superimposed in the target plane. This leads to unwanted interference effects when using coherent beam profiles [16]. Therefore, they can only be used for partially coherent or incoherent beams.

An advantage of refractive beam shaping elements is their typical high efficiency and the wide useable wavelength range. So far, there are limitations in manufacturing, as the radius of curvature at the surface cannot be too small. As a result, not every desired light distribution can be generated by refractive elements. Furthermore, the achievable spot sizes of refractive beam shapers are above the Gaussian diffraction limit [13].

To realize spot sizes close to the Gaussian diffraction limit, diffractive beam shapers have already been utilized [17,18,19,20,21,22]. The described concepts based on the requirement of converting the incoming laser beam into a 1-D sinc(x) or 2-D Bessinc(r) intensity distribution to generate a one-dimensional or round Top-Hat profile in the focal plane. This requirement comes from the reciprocal Fourier relationship between the input plane and the desired far-field or focal-plane profile [23,24]. For instance, it was shown that a binary diffraction grating with very low periodicity and a central π-phase reversal can satisfy this requirement, leading to energy efficient and flexible elements [17,18,19,20,21,22]. However, for many applications in laser material processing, a square-shaped diffraction-limited Top-Hat beam profile is needed.

In this study, we present a design concept for a binary diffraction grating with very low periodicity and a central π-phase reversal for the generation of a square-shaped diffraction limited Top-Hat beam profile. The calculated surface structure of the so-called FBS beam shaper is transferred into a fused silica glass substrate using a combination of microlithography technologies. Finally, the square Top-Hat beam profile generated by the manufactured FBS beam shaper is evaluated.

## 2. Design Method of Fundamental Beam Mode Shaper 

Based on the reciprocal Fourier relationship, a square homogeneous light distribution in the far field requires an sinc(x,y) = sinc(x) ∙ sinc(y) amplitude distribution at the input plane. The sinc(x,y) function is real and shows a modulation of amplitude with zeros and a periodic change of sign [23,24]. The change of sign corresponds to a binary change in phase from 0 to π, and vice versa. Due to the high efficiencies required in laser material processing, a modulation of the amplitude of laser radiation by an optical element is not acceptable for generating a square Top-Hat profile. However, by combining a Gaussian amplitude distribution g(x,y) with the phase distribution of the sinc(x,y) function, the ideal function at the input plane can be approximated as described below.

**Figure 1:**
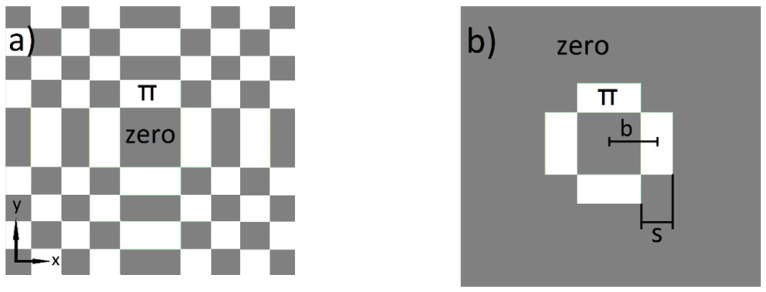
(**a**) Principle phase distribution of sinc(x,y) function; (**b**) First inner phase change of sinc(x,y) function (coordinate origin in the center).

In Figure 1a, the principle binary phase distribution of the sinc(x,y) function is shown. By neglecting the boundary area and concentrating on the first inner change of phase, the binary phase distribution of the sinc(x,y) can be approximated as shown in Figure 1b.

The area of the first inner change of phase can be described as follows:

phase(x,y) = π ∙ (rect((x ± b)/s) ∙ rect(y/(2b − s)) + rect((y ± b)/s) ∙ rect(x/(2b − s)))(1)

As mentioned before, in laser material processing, collimated Gaussian amplitude profiles g(x,y) with a constant phase front are typically used. These profiles can be described by:

g(x,y) = exp((−(x^2^+y^2^))/ω^2^) (2)

where ω is the beam radius when amplitude has fallen to 1/e of the maximum value. In combination with Equation (1), a local phase change of the Gaussian amplitude profile g(x,y) can be realized leading to the complex field distribution g_fbs_(x,y), which corresponds to an approximated sinc(x,y) function:

g_fbs_(x,y) = exp((−(x^2^+y^2^))/ω^2^) ∙ exp(−i ∙ phase(x,y)) (3)

**Figure 2:**
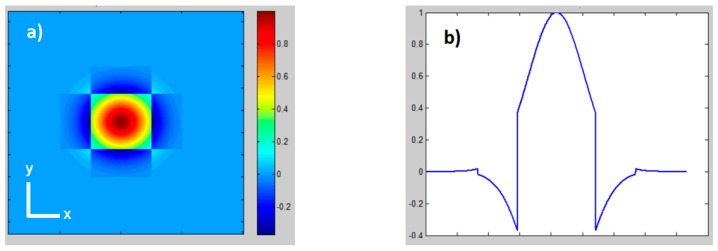
(**a**) Amplitude distribution of g_fbs_(x,y); (**b**) Line scan of amplitude distribution of g_fbs_(x,0), coordinate origin in the center.

The amplitude distribution of g_fbs_(x,y) is shown in Figure 2.

The resulting complex field distribution in the far field or output plane G_fbs_(u,v) can be calculated by the execution of the Fourier transformation g_fbs_(x,y) of the input plane. In an optical setup, the required Fourier transformation can be realized by a focusing lens [23], leading to the resulting complex field distribution in the focal plane.

For the analytical Fourier transformation of g_fbs_(x,y), it should be considered that the phase changes from 0 to π and vice versa, g_fbs_(x,y) just leads to a change of amplitude sign.

By the definition of phase(x,y)/π as the Fundamental Beam Mode Shaping function, fbs(x,y), the resulting sign for the modulation of amplitude is given by:

sign(x,y) = 1 − 2∙fbs(x,y)(4)

Then, by multiplying the amplitude modulation function sign(x,y) with this Gaussian beam profile g(x,y), the approximated sinc_fbs_(x,y) function can be generated:

sinc_fbs_(x,y) = g(x,y) – 2 ∙ fbs(x,y) ∙ g(x,y) (5)

This leads to the same amplitude distribution as g_fbs_(x,y), as shown in Figure 2.

Finally, the Fourier transformation TH(u,v) of sinc_fbs_(x,y) can be described by Equation (6), with the relations provided by Equations (7) and (8):

TH(u,v) = G(u,v) – 2 ∙ FBS(u,v) ∗ G(u,v)(6)

G(u,v) = ω^2^ ∙ π ∙ exp(−(u^2^ + v^2^ ) ∙ ω^2^ ∙ π^2^) (7)

FBS(u,v) = s ∙ (2 ∙ b − s) ∙ (a(u,v) + b(u,v))(8)

where:

a(u,v) = sinc(u ∙ s) ∙ sinc(v ∙ (2 ∙ b − s)) ∙ 2 ∙ cos(2 ∙ π ∙ b ∙ u)(9)

b(u,v) = sinc(v ∙ s) ∙ sinc(u ∙ (2 ∙ b − s)) ∙ 2 ∙ cos(2 ∙ π ∙ b ∙ v)(10)

**Figure 3:**
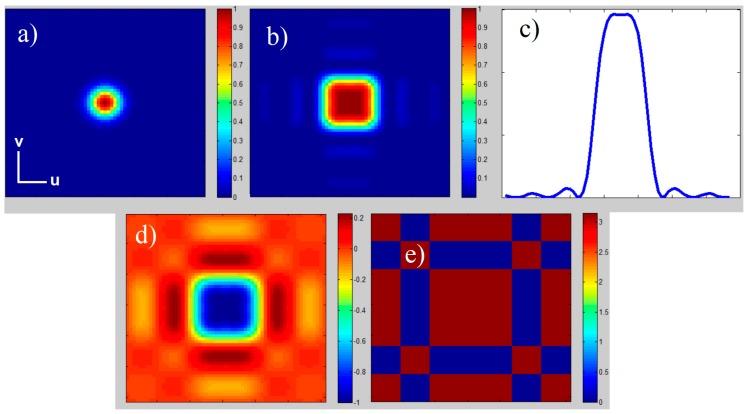
Theoretical simulations, with relation s = ω and b = 1.5 s: (**a**) Gaussian intensity distribution |G(u,v)|^2^; (**b**) Top-Hat intensity distribution |TH(u,v)|^2^; (**c**) Line scan Top-Hat intensity distribution |TH(u,0)|^2^; (**d**) Top-Hat amplitude distribution of TH(u,v); (**e**) phase distribution of TH(u,v); coordinate origin in the center.

As shown in Equation (6), TH(u,v) is a real function, and the amplitude distribution depends on the combination of the parameters b, s and ω. With the relation b−s/2 = s = ω, a square Top-Hat profile can be generated, as shown in Figure 3.

By applying the fbs(x,y) function on the input beam profile, the beam diameter in far-field just slightly increases. The ratio of the edge length of the Top-Hat intensity profile |TH(u,v)|^2^ at 1/e² and the unshaped diffraction limited Gaussian intensity profile |G(u,v)|^2^ at 1/e² diameter is about ~1.5. Top-Hat generation using the fbs(x,y) function shows high efficiency (>95%) and a homogeneous phase distribution within in the Top-Hat area.

The calculated phase distribution of the fundamental beam mode shaper (Equation (1)) corresponds to an optical element with just two different height levels. When a Gaussian laser beam passes through such an optical element, the height levels causes the desired phase change of π for a part of the beam leading to an inverted amplitude sign.

The height profile of the fundamental beam mode shaper can be transferred in fused silica substrates (refractive index n = 1.4607 at a wavelength λ = 532 nm) by using microlithography, reactive ion etching (RIE) and ion beam etching (IBE) [25]. The required etch depth z for achieving the phase changes φ of π can be calculated by Equation (11):

z = λ/(2 ∙ (n−1))(11)

For the parameters described above, an etch depth of 577 nm can be calculated.

## 3. Manufacturing Method for FBS Beam Shaper

Ion-Beam-Etching (IBE) smooth surfaces with high optical quality. However, due to the poor selectivity of this etching process, mask layers of few microns thickness are needed [26]. Thick photoresist layers require a long hard bake time at high temperatures (200 °C) to withstand the IBE process. Compared to IBE, the etching rates of plasma-based Reactive-Ion-Etching (RIE) are significantly higher, which leads to shorter process times. The high selectivity of the process also allows the use of thin mask layers. However, reactive ion-etched areas show a relatively high nano-surface roughness. Therefore, a process technology was developed that combines the advantages of RIE and IBE [27].

**Figure 4:**
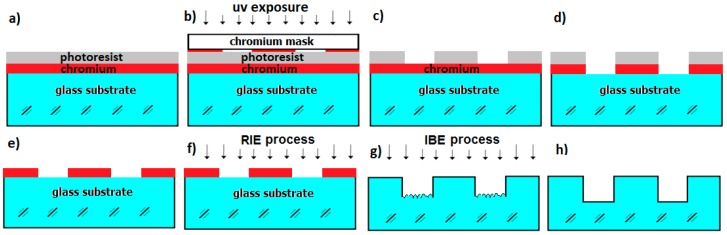
Principle of lithographic structuring of FBS glass substrate: (**a**) Deposited 100 nm chromium layer and 1.4 µm photoresist layer; (**b**) Photolithographic contact UV-exposure using chromium coated mask; (**c**) Developed photoresist layer; (**d**) Wet chemical etched chromium layer; (**e**) Removed photoresist structure; (**f**) RIE structuring of glass substrate; (**g**) Reduction of nano roughness using IBE process; (**h**) Final structured FBS element.

The FBS beam shaper was fabricated by depositing a 100 nm chromium layer with magnetron sputtering and a 1.4 µm photoresist AZ5214 layer on fused silica glass substrates (Figure 4a). After photolithographic contact UV exposure using a chromium-coated mask (Figure 4b) and development of the photoresist (Figure 4c), the chromium film on glass substrate was patterned by wet chemical etching in HClO4/Ammonia-Cer-Nitrate solution (Figure 4d). Thereby, the masking of the chromium-coated mask corresponds to the desired lateral structure of the FBS element.

After the patterning of the chromium layer, the resist was removed by using AZ100 remover (Figure 4e). The thin chromium film acts as a very stable masking layer on the glass substrate for the subsequent RIE process (Figure 4f). The RIE process caused unwanted surface nano roughness, which could be significantly reduced by an additional IBE process (Figure 4g,h). A more detailed description of both etching process and the reduction of nano roughness is covered below:

RIE was performed in a capacitively coupled plasma of a parallel plate reactor, which operates at a radio frequency of 13.56 MHz. Pure SF6 and gas mixtures of SF6/Ar were used as process gases, which generate plasmas with fluorine radicals. The reaction products of these radicals with glass (e.g., SiF4, SO2F2) are volatile and can be evacuated by the vacuum pump. The chemical reactions at the etched surface are influenced by several process parameters. They depend on gas mixture, flow rate, plasma power, temperature, and chamber pressure [28,29,30,31,32,33]. These parameters influence the etching rate, selectivity, homogeneity and anisotropy of the process.

To achieve high etching rates, a combination of high power, high gas flow and low chamber pressure is required. The typical chamber pressure varies between 0.1 and 10 Pa. The power ranges from 50 to 300 W and the gas flow typically is between 20 sccm and 100 sccm. Depending on process and glass type, the etching rates range from 10 nm/min up to 500 nm/min [30]. Non-volatile reaction products like sodium fluorides contaminate the surface during RIE and lead to the formation of rough surfaces. The effect can be reduced by mixing the reactive gas with an inert component like Ar, which facilitates the physical removal of contaminations [31,32].

The etch rate and surface roughness of fused silica as function of various RIE process parameters (plasma power, process pressure, flow rate) were investigated for different mixtures of SF6 and Ar. For the measurement of etching depth and surface roughness, a Dektak III Stylus Profiler (Bruker, Billerica, MA, USA) was used. The results of the etching process are depicted in Table 1.

As expected, the etching rate increases with power and decreases with chamber pressure (see Table 1). It also increases with the argon fraction and ranging from 14.6 nm/min to 64.4 nm/min. The average surface roughness after the etching process was between 4 nm and 12.1 nm. For this parameter, no correlation with the etching rate was observed. The experiments described in detail in [27] showed that the best surface roughness can be achieved with a RIE process that applies 20 sccm SF6, 150 W plasma power and 60 mTorr chamber pressure. These parameters were employed for the FBS fabrication.

**Figure 5:**
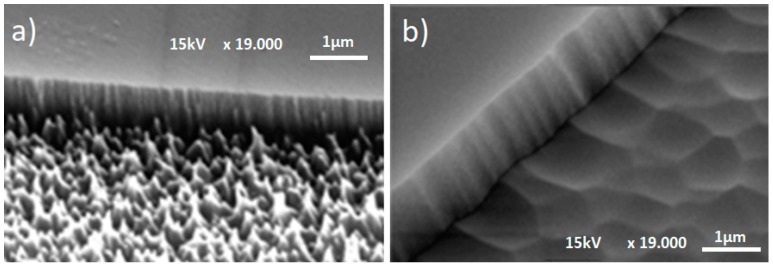
SEM pictures of RIE-etched surface (SF6-Plasma with 20 sccm flow-rate, 270 W, 30 mTorr): (**a**) Before and (**b**) After 15 min of IBE treatment (Ar-ion beam, 1000 V acceleration voltage, 80 mA beam current).

Using SEM, the RIE etched surfaces were also characterized. This allowed to observe a nano roughness, which was detectable with the Dektak III Stylus Profiler (see Figure 5). The nano roughness of the RIE etched areas (Figure 5a) can reduce the optical performance of the FBS, by diffusing the incoming light. Pretests indicated that the IBE process can produce significant smoothing of the fused silica surface after 15 min process time (Figure 5b).

To smooth the remaining surface nano roughness, a Kaufman ion source (NTG Neue Technologien GmbH & Co. KG, Gelnhausen, Hesse, Germany) with 10 cm beam diameter was used. Beam voltages in the range from 600 V to 1200 V were applied. The beam current was kept at 80 mA. With increasing beam voltage, the etching rate increases from about 10.6 nm/min to 15.1 nm/min. This smoothing effect is caused by the strong dependence of the ion beam etching rate on the incident beam angle, as reported in [34,35,36,37,38,39]. Under perpendicular alignment of the beam to the substrate surface, tips are ablated faster than the even areas. This allows smoothing the fused silica surface.

As indicated in Table 2, the surface roughness (Dektak measurement) does not change with beam voltage. The roughness Ra (measured in a scan field of 0.5 × 0.5 mm²) indicates values between 3 nm and 6 nm and is still comparable to that of non-etched areas with optical surface quality.

**Figure 6:**
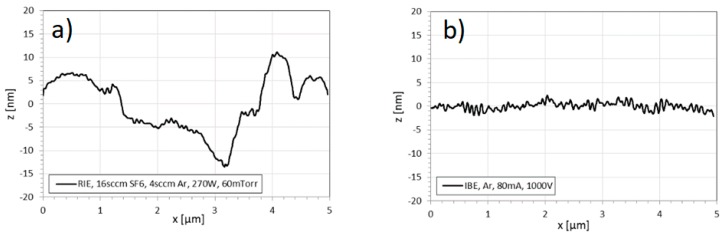
AFM measurements of surface nano roughness (**a**) after RIE, (**b**) after RIE/IBE.

For the measurement of the micro roughness introduced by the RIE process, the AFM method was used. Figure 6 shows representative AFM measurements of the processed substrate surfaces after RIE (Figure 6a) and after RIE/IBE post processing (Figure 6b) in a scan field area of 5 × 5 µm². The results demonstrate a significant improvement of the nano roughness due to the IBE process, with a reduction of Ra from 7 nm to less than 2 nm.

The etching depth is one important parameter with respect to the optical performance of the FBS. The depth can be measured by using a Dektak III Stylus Profiler with a measuring accuracy of ±4 nm. Measurements before and after the IBE post processing demonstrate that the etching depth changes significantly. The increase depends on the process parameters and the etching time (etch depth), of the RIE process. The faster IBE ablation of rough etched areas compared to non-etched surface regions leads to this change of the measured depth.

For the RIE process with 60 mTorr chamber pressure, 150 W plasma power and 20 sccm SF6 flow-rate, two series of fused silica samples were etched with depths varying between 150 and 1200 nm. The samples feature binary lines and spaces structures with 40 µm grating period. The increase of depth was measured after 15 min of IBE post processing (beam voltage: 1000 V, beam current: 80 mA).

**Figure 7:**
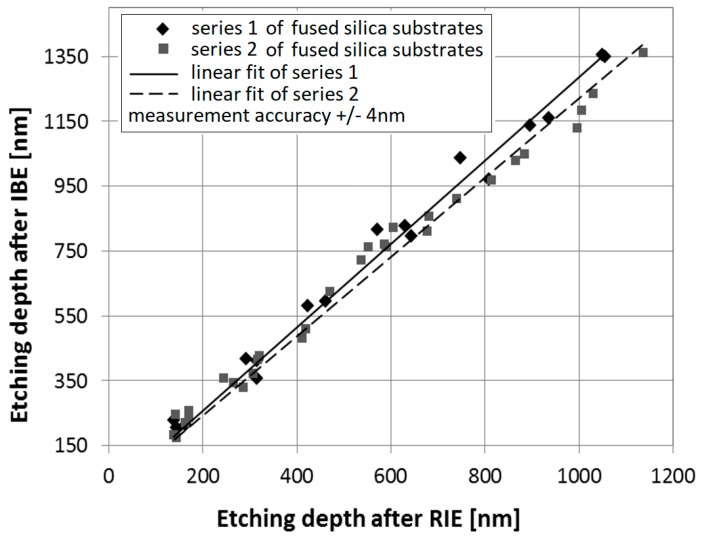
Linear relation between the etching depth after RIE (d_RIE_) and after IBE post processing (d_IBE_).

The experiments demonstrate a linear relation between the etching depth after RIE and after IBE post processing, as shown in Figure 7. Two different measurement series were carried out. The RIE and IBE etching depth was measured with Dektak III with a measuring accuracy of ±4 nm. With the stated RIE parameters, the depths ratio d_IBE_/d_RIE_ amounts to roughly 1.2.

This ratio has to be taken into account when planning the combined fabrication process of RIE and IBE in order to achieve the required depth (phase change) of the patterned areas. 

## 4. Optical Evaluation of Manufactured FBS Element

**Figure 8:**
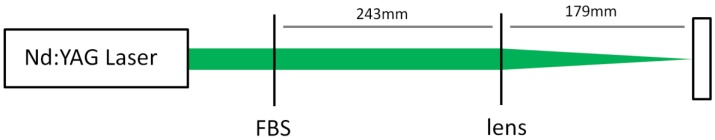
Experimental setup used for characterizing the produced Top-Hat profiles, with a frequency doubled cw Nd:YAG laser (532 nm wavelength, output power ~10 mW).

The experimental setup for reshaping a round Gaussian input beam into a square Top-Hat beam profile using the binary FBS phase plate is shown in Figure 8. A frequency doubled cw Nd:YAG laser with 532 nm emitting wavelength (output power ~10 mW) and collimated beam was focused by a lens with f = 150 mm on a WinCam CCD camera (DataRay, Redding, CA, USA) to record the resulting beam profiles with and without introducing an FBS beam-shaping element into the beam path.

Before introducing the beam shaper into the beam path, the beam profile at the FBS plane was measured, delivering a mean beam diameter of Gaussian beam of 1.1 mm and an ellipticity of 0.98. At the plane of the focusing lens, 243 mm behind the plane of FBS, the beam diameter was 1.2 mm with an ellipticity of 0.97.

**Figure 9:**
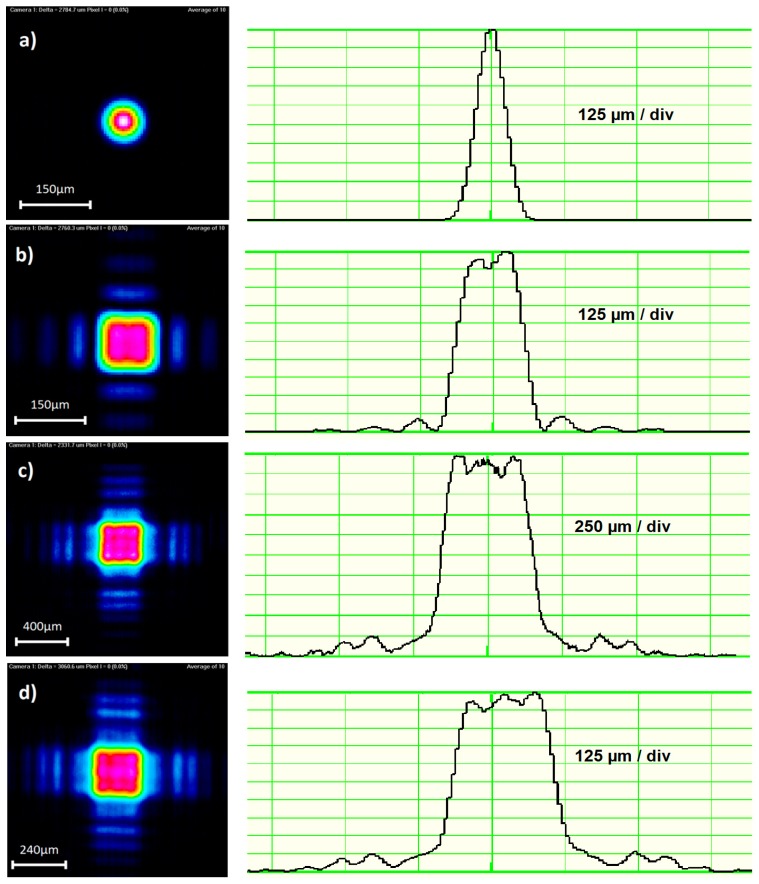
Measurement of generated beam profiles (**a**) Focused Gaussian profile at beam waist; (**b**) zero-order Top-Hat profile; (**c**) −1 order Top-Hat profile; (**d**) +1 order Top-Hat profile.

The beam waist of the focused beam was measured at the position z = 179 mm, showing a beam diameter of 98 µm (Figure 9a). By introducing the FBS element into the beam path, the original round Gaussian beam was transformed into a square Top-Hat beam profile with an edge length of 153 µm as shown in Figure 9b. The Top-Hat profile at the location of Gaussian beam waist will be defined as zero-order Top-Hat. By analyzing the beam profiles along the propagation path behind the focusing lens, further Top-Hat beam profiles could be detected. At 7.9 mm before the zero-order Top-Hat, the so-called minus first-order Top-Hat with an edge length of 400 µm was observed (Figure 9c). At 4.8 mm behind zero-order Top-Hat, the so-called plus first-order Top-Hat, with an edge length of 240 µm, could be seen (Figure 9d).

These experimental results confirm the principle design concept for the FBS beam shaper for the Top-Hat generation presented in this study. However, the measured +/− first order Top-Hat profiles behind and in front of the focal plane are not described by the design concept. Therefore, further investigations are necessary to understand these effects.

The generated zero-order Top-Hat (Figure 9b) is about 1.5 times bigger as the unshaped Gaussian intensity profile as expected. However, it exhibits a not completely flat center part. The intensity modulation is roughly ±5%. Furthermore, the energy fringes are roughly twice higher in comparison to the theoretical results shown in Figure 3c, leading to a lower efficiency of Top-Hat generation.

These energy fringes could cause unwanted heat transfer into the material. The authors got the feedback from an industrial partner that for a special semiconductor process the heat generated by these energy fringes lead to unwanted damage of the material. It depends on the application and the certain parameter set of pulse energy, pulse duration, material and wavelength if the beam shaping result generated by the FBS element is suitable. However, these energy fringes are typically far below the ablation threshold and are uncritical for the process [10].

Reasons for these deviations from the ideal Top-Hat profile could be non-ideal phase and amplitude distribution of used Gaussian beam, differences between theoretical and produced FBS surface, and aberration effects of focusing optic. Here, it will be necessary to carry out further investigations to understand the influence of theses parameter on the beam-shaping result.

## 5. Conclusions

In this study, a design concept for a binary phase distribution with low periodicity and a central π-phase reversal is presented. This design transforms a Gaussian beam profile in the input plane into a square-shaped diffraction-limited Top-Hat beam profile in the far field. It was shown that 95% of the input energy can be transferred to the Top-Hat profile with a homogenous phase distribution. The calculated phase distribution of the so-called fundamental beam mode shaper (FBS) corresponds to an optical element showing just two different height levels. The height profile of the FBS is transferred in fused silica substrates by using microlithography, reactive ion etching (RIE) and ion beam etching (IBE). The RIE process generates an undesired nano surface roughness; therefore, IBE is used to smooth the surface. The experiments demonstrate a linear relation between the etching depth after RIE and IBE, leading to a depths ratio d_IBE_/d_RIE_ of roughly 1.2. The optical evaluation of the manufactured FBS beam mode shaper confirms the presented concept design. In addition, it is shown that besides the Top-Hat profile in the focal plane (zero order), further Top-Hat profiles (+/− first order) occur along the propagation path behind the focusing lens. Further investigations are necessary to understand these effects.

## Figures and Tables

**Table 1:** RIE parameters and resulting etching rates and average surface roughness Ra.

No.	SF6 (sccm)	Ar (sccm)	P (W)	p (mTorr)	Etching Rate (nm/min)	Average Ra (nm)
1	20	0	270	60	36.8	6.7
2	20	0	270	30	42.9	12.1
3	20	0	150	60	14.6	4.0
4	20	0	150	30	22.7	7.7
5	16	4	270	60	44.3	5.4
6	16	4	270	30	55.3	4.9
7	16	4	150	60	20.0	5.7
8	16	4	150	30	27.8	4.7
9	10	10	270	60	49.5	5.4
10	10	10	270	30	64.4	7.0
11	10	10	150	60	27.9	8.7
12	10	10	150	30	34.2	10.1

**Table 2:** Surface roughness Ra (Dektak measurement) after IBE as function of beam voltages.

Beam Voltage (V)	Ra (nm) (non-etched area)	Ra (nm) (RIE/IBE etched area)
600	3.7	5.9
800	4.4	4.6
1000	1.9	3.4
1200	4.3	5.1
